# Dynamical Symbiosis of Solar Cell and Memristor

**DOI:** 10.1021/acsenergylett.6c00713

**Published:** 2026-05-08

**Authors:** Jitendra Kumar, So-Yeon Kim, Gonzalo Rivera-Sierra, Juan Bisquert

**Affiliations:** Instituto de Tecnología Química (ITQ), Consejo Superior de Investigaciones Científicas-Universitat Politècnica de València (CSIC-UPV), Valencia 46022, Spain

## Abstract

Memristive devices have attracted significant attention due to their nonlinear dynamics, analog tunability, and ability to emulate synaptic functions. When combined with energy-harvesting components, memristors offer an opportunity to realize self-powered physical AI systems. We demonstrate a solar cell-driven perovskite memristor architecture, which is suitable for autonomous physical AI platforms for light-driven computation. In this system, incident light is directly converted into electrical stimuli by integrated solar cells, which modulate the conductance states of memristors. These conductance changes can encode computational states and learning behaviors, enabling direct processing of optical information without intermediate digital conversion. Such a light-driven memristive system enables simultaneous sensing, energy harvesting, memory storage, and computation within a unified physical structure.

## Graphical abstract

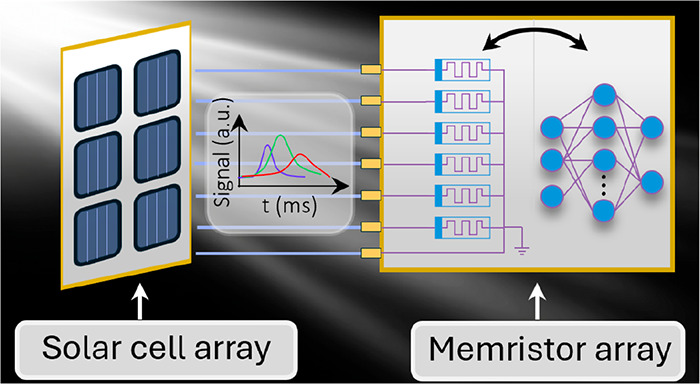

Present day artificial intelligence (AI) technologies largely depend on software-defined architectures operating on conventional electronic processors.^1–4^ While such systems have demonstrated remarkable performance in data-intensive tasks, they face inherent limitations related to energy consumption,^5–7^ latency,^8–11^ scalability,^12,13^ and physical disconnection from real-world stimuli.^14^ Current AI architectures rely heavily on centralized processing,^15–18^ frequent data movement between memory and computation units, and continuous power supply, leading to inefficiencies that constrain their deployment in resource-limited or edge environments.^19,20^ Moreover, software-based AI lacks intrinsic adaptability to physical signals, requiring extensive preprocessing to interface with sensory data such as light, temperature, or mechanical stimuli.

Physical AI has emerged as a promising paradigm that embeds intelligence directly into material systems and devices, enabling computation through physical processes rather than abstract digital operations.^21–24^ Physical AI takes advantage of physical processes to perform computation, instead of simulating everything digitally. By exploiting the natural dynamics of materials, physical AI offers advantages including massive parallelism,^25,26^ real-time responsiveness,^9,27^ and tight coupling between sensing, memory, and computation.^19,28–30^ Such systems can process information in situ, reducing data transfer overhead and enabling autonomous operation in distributed and edge scenarios. Importantly, physical AI provides a pathway toward neuromorphic and brain-inspired computing, where learning and inference occur through local, adaptive physical states in a more efficient way, requiring much less energy as demanded by conventional software defined AI platforms.

To deal with the giant energy requirement of current AI systems, Fadi et al. built a robust AI chip using memristors in a binarized neural network that runs directly from a tiny solar cell and performs the task of image classification.^31^ Their experiment showed that, under high illumination (≈8 suns) their solar cell driven device shows the comparable performance to that of a stable, regulated power source, whereas it maintains the functionality even at extremely low light intensity as low as 0.008 suns.^31^ Xiaoyang et al. designed a self-powered artificial retina system by integrating a silicon solar cell and halide perovskite memristors to improve the performance of 784 × 25 × 10 three-layered artificial neural networks for the task of image recognition.^32^

These works demonstrate the potential of integrating memristors and solar cells into a unified platform, enabling a transition from software-defined AI to physically realizable, energy-autonomous intelligent systems. The approach of combining energy harvesting and computation within the same device transforms edge hardware from passive sensors into active computational units and overcomes the von Neumann bottleneck through in-sensor computing. Information processing is performed directly at the light–matter interface via parallel analog operations, avoiding costly data digitization and transfer. As a result, latency and power consumption are significantly reduced, supporting real-time, adaptive neuromorphic computation in resource-constrained environments.

While these works provide a direction for self-powered AI, further work is required to understand the emergent coupling dynamics between photovoltaic cells and memristor units to utilize the full potential of their dynamical symbiosis. A sound understanding of these interactions will enable the development of systems that can mimic the metabolic homeostasis of the brain. Just as the biological brain modulates synaptic activity to survive metabolic scarcity, this memristor - solar cell coupling can facilitate computational homeostasis, allowing hardware to autonomously adjust its cognitive workload in real time, based on available ambient energy.

To investigate the dynamics of a coupled memristor–solar cell system, we integrated a volatile perovskite memristor with a silicon solar panel and experimentally demonstrated the switching behavior of the memristor driven by the solar cell. Since the solar cell cannot reverse the polarity of its output voltage, employing a volatile memristor enables setting and resetting of the memristor through the adjustment of the incident light intensity alone. The details of device preparation and measurement can be found in the experimental section. In this configuration, the perovskite memristor operates as a resistive switching element, while the Si solar cell serves as the system driver. Illumination of the solar cell generates a potential difference between its selective contacts. In the coupled memristor–solar cell system this potential difference is observed as the operating point of the complete systems, the operating point voltage (*V*_*op*_) is determined by the intersection of the memristor’s load line and the current–voltage (IV) characteristics of the solar cell.

Figure 1 illustrates the dynamics of the operating point of the coupled system under three different illumination conditions. In general, if the solar cell can produce voltages exceeding the memristor threshold voltage (*V*_*th*_) then the incident light intensity can be adjusted to set the operating point either above or below the memristor’s threshold. In this study, we used a toy solar panel composed of several small silicon solar cells. Under illumination, when the operating point of memristor-solar cell coupled system falls below (*V*_*th*_), the memristor remains in the high-resistance state (HRS), and the system voltage is stable. However, when increased illumination shifts the operating point above (*V*_*th*_), the system initially starts at the intersection of the solar cell’s IV curve corresponding to the given illumination and the memristor’s load line corresponding to the HRS. As the memristor conductance subsequently evolves, the operating point dynamically traces the solar cell IV curve and ultimately settles at the intersection of the solar cell’s IV characteristics and the memristor’s final low-resistance state (LRS). The evolution of this dynamic system is schematically indicated by the gradient-colored arrow in Figure 1a.

**Figure 1:**
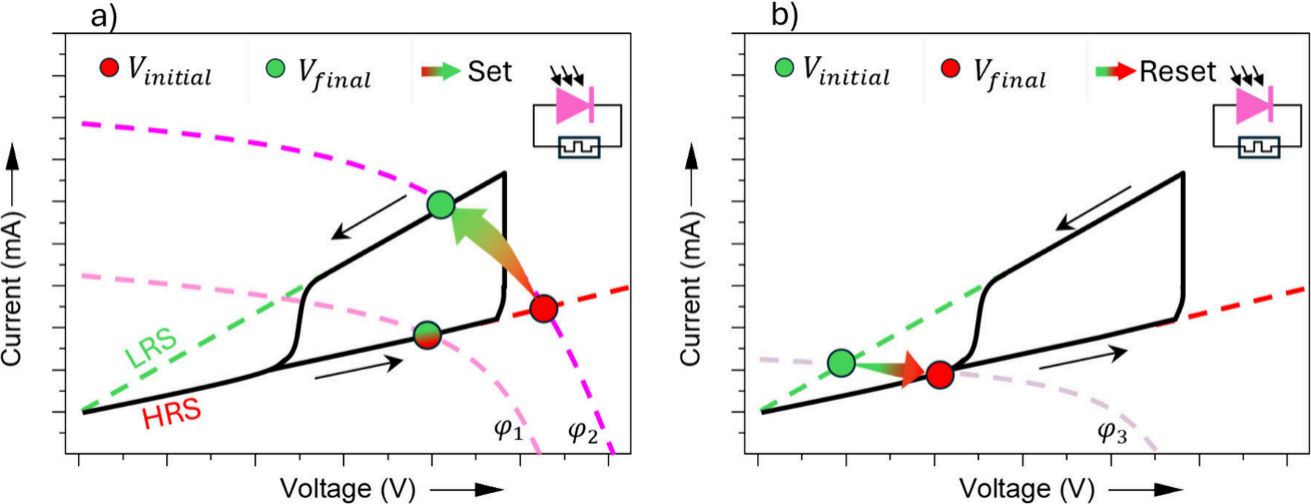
Dynamics of coupled memristor-solar cell system. φ_1_, φ_2_, φ_3_ are different light intensities driving the coupled memristor-solar cell system, in this case the memristor is a volatile memristor. Curves with dashed lines (dark to light magenta color) represent the current voltage (IV) curve of the solar cells under different light illumination conditions. The black solid curve represents the IV curve of the volatile memristor. The gradient-colored arrow shows the transition of operating point of coupled system as the memristor makes transition from a) high resistance state (HRS) to low resistance state (LRS) (set) or b) LRS to HRS (reset).

Memristors are state-dependent systems with dynamical behavior. Therefore, if a memristor is programmed into the LRS by an earlier light pulse, after removing the light pulse it begins to relax toward the HRS due to its volatile nature. Now, if a subsequent, low-intensity light stimulus is applied immediately after setting the memristor into LRS (before memristor naturally returns to the HRS due to its volatile nature), the system will show a transition of the operating point from LRS toward HRS of the memristor as shown schematically in Figure 1b. The low-intensity condition is defined such that the operating point remains below the memristor’s switching threshold even when the device is in the HRS.

Figure 2a shows the experimental IV curves of a solar panel module at the peak light intensity used for recording the light intensity vs current (I-ϕ) and light intensity vs voltage (V-ϕ) curves. A green LED source from Autolab (AUT.LED.LDC530.S) is used for all the measurements. Figure 2b shows the IV characteristics of perovskite memristor. To record the memristor IV a cyclic voltage sweep (0→ 1.2→ -1.2→ 0) is used. The voltage scan rate for recording this IV curve is fixed to 2 V/s (0.42 Hz). Comparing these two data sets reveals that solar panel’s open-circuit voltage exceeds the memristor’s switching threshold, this enables the coupled system to operate and display memristor’s switching without external bias. Memristor’s IV curve shows that the memristor displays capacitive hysteresis in the forward bias, whereas it shows inductive hysteresis in the reverse bias.

**Figure 2:**
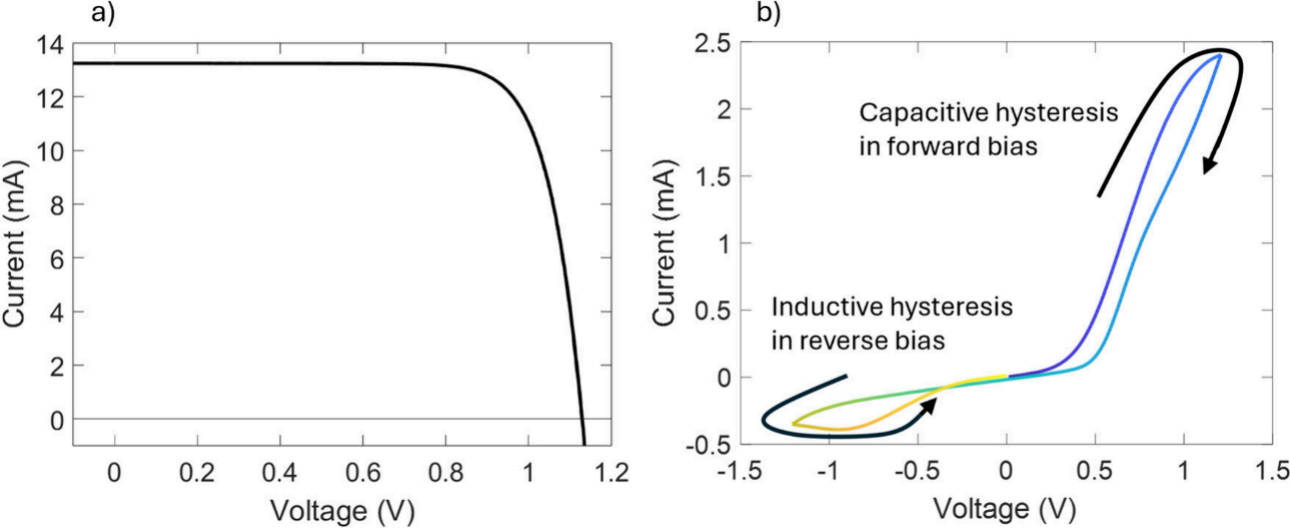
a) Experimental IV curve of the solar cell under illumination of green light. b) IV curve of the memristor at scan rate 2 V/s.

In halide perovskite based memristors both the inductive and capacitive hysteresis are well-known in the literature.^33,34^ The primary mechanism for capacitive hysteresis is the ion migration and double layer formation, which also leads to the conducting filament formation between the electrodes of the device and becomes the reason for resistive switching in memristors.^35^ Capacitive dynamics mainly determine how quickly the voltage across the active switching region reaches the threshold required for state change, thereby affecting switching speed, pulse response, and apparent hysteresis behavior. Whereas the inductive hysteresis is the result of ionic electronic coupling which leads to the slow ion-modulated electronic recombination, lattice relaxation, and trap-mediated delay.^36^ Inductive dynamics mainly influence transient current and voltage overshoots; they can cause ringing or unwanted spikes that may unintentionally trigger switching, especially in threshold-sensitive devices. The interaction of these effects strongly impacts device stability, repeatability, and programmability, particularly for analog conductance tuning and neuromorphic applications where controlled and gradual resistance updates are essential. Inductive hysteresis can be exploited for learning because it reflects delayed, history-dependent conductance modulation that enables synaptic plasticity.^37,38^ Therefore, the reverse bias case (solar cell’s cathode connected to the memristor’s anode) is further studied to investigate the mechanism of learning using light as the only energy source.

Figures 3a and 3b show the time-dependent current and voltage responses of the coupled system to applied light pulses, respectively. The light intensity scale is normalized here, and the absolute peak power density is not critical, provided that the solar module generates a voltage exceeding the switching threshold of the memristors. The I–V curve in Figure 3c is derived from the current and voltage data presented in Figures 3a and 3b by correlating the two data sets by matching the corresponding light intensities from two measurements. IV and I-ϕ characteristics in 3c, and 3d demonstrate direct light-driven modulation of memristive conductance states.

**Figure 3:**
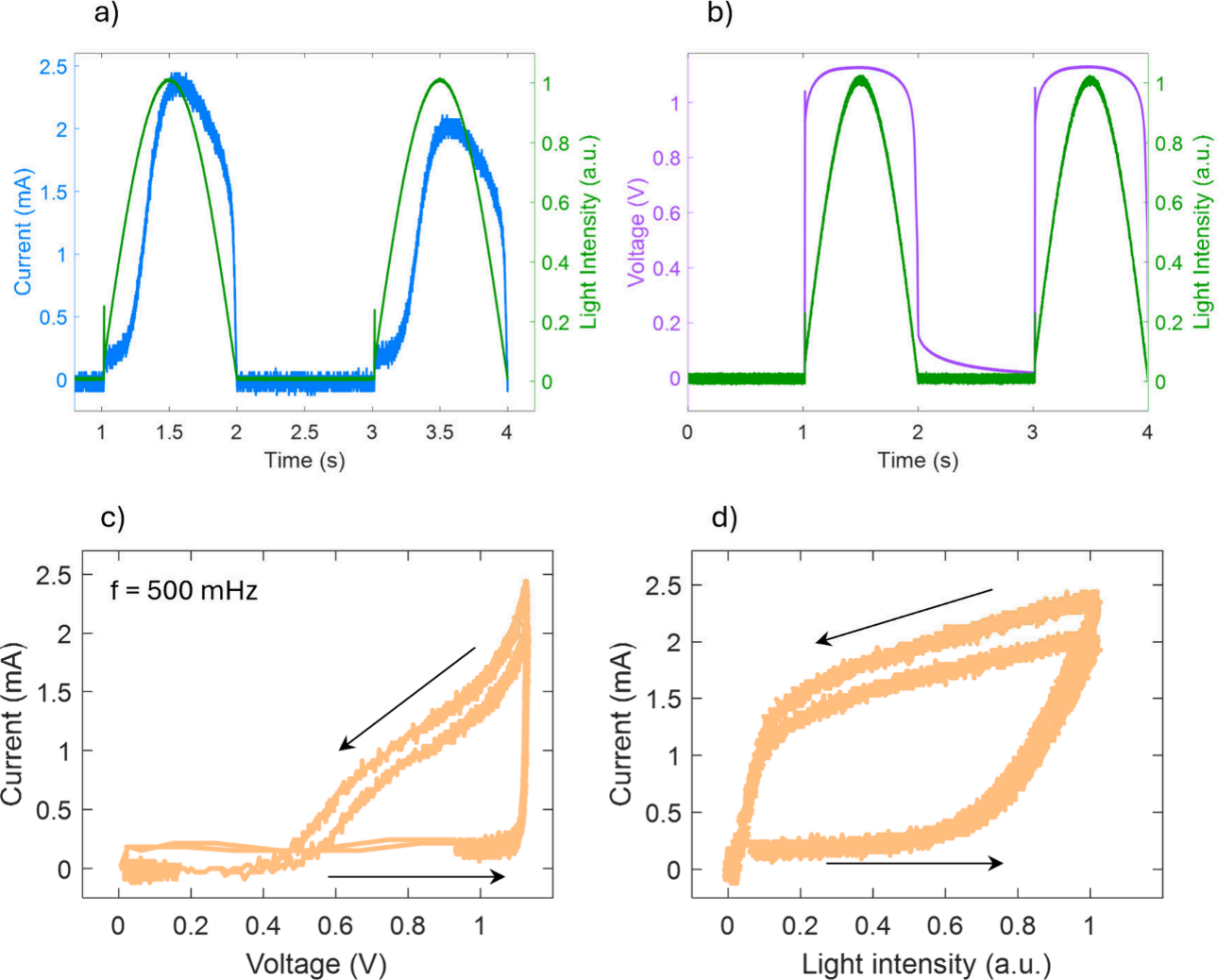
a) Time dependent current vs light intensity and b) voltage vs light intensity, c) voltage (photovoltage) vs current (photocurrent) charecteristis of the coupled system, d) light intensity vs current charecteristics.

Figure 4 shows the modulation frequency dependent dynamics of the coupled system. As shown the system exhibits a transition from the low-resistance state (LRS) to the high-resistance state (HRS) at approximately 1.1 V. At the scan rate used for the demonstration, the memristor predominantly displays inductive hysteresis. However, at very low modulation frequencies (<500 mHz) a capacitive current component becomes apparent as shown in Figure S4. This capacitive current originates from the memristor’s large internal capacitance, as commercial Si solar cells lack significant capacitance and provide a nearly instantaneous response.

**Figure 4:**
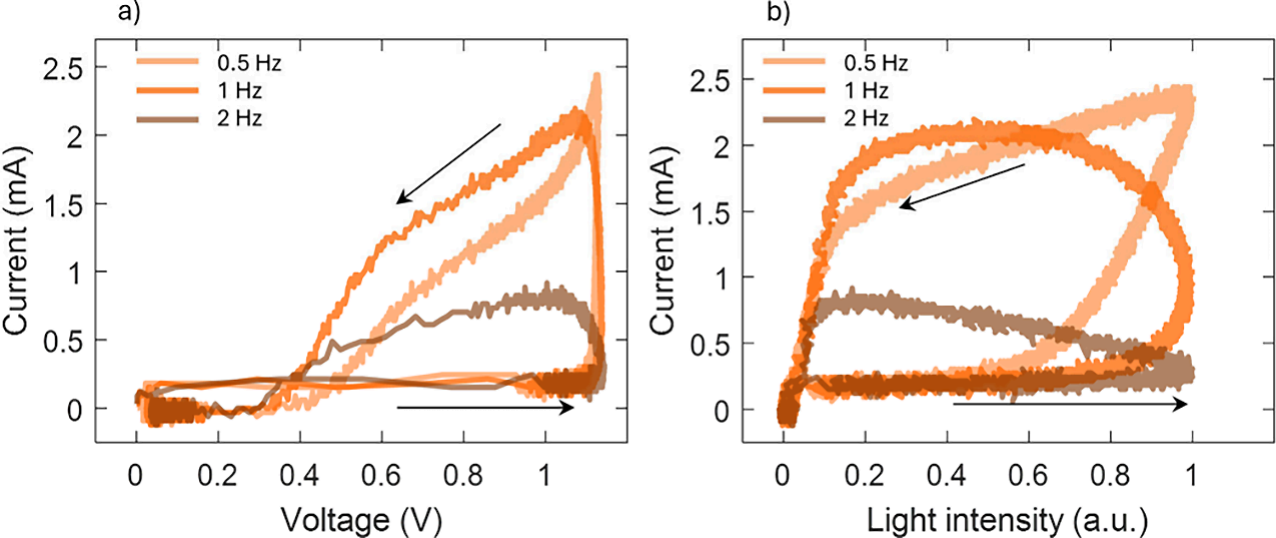
a) IV curves of solar cell-memristor coupled system at different modulation frequencies. b) Corresponding light intensity vs current curves.

To perform the V-ϕ or I-ϕ measurements, the incident light intensity on the solar cell is varied sinusoidally. At relatively high modulation frequency, as the light intensity is increased from zero the voltage rapidly rises from 0 to approximately 1 V and then changes only little. This behavior (almost constant voltage at higher light intensity) arises partly from the logarithmic dependence of the solar-cell voltage on light intensity and partly from changes in the memristor conductance. The conductance-induced change in voltage (the drift of the operating point) occurs in the opposite direction to the voltage change caused by variations in light intensity. Thereby, effectively reducing the change in voltages above 1 V, this explains extremely sharp transitions from LRS to HRS when driving by light as compared to slow transition from HRS to LRS when memristor is driven by voltage, see Figure 2b and Figure S4a.

Over the full illumination cycle, the current remains attenuated at high modulation frequencies (for example at 2 Hz). Since the cycle period is shorter than the inductive time constant, the current fails to track the photovoltage generated by the solar cell. When the modulation frequency is reduced from 2 to 1 Hz, the system has more time to respond, showing a larger inductive hysteresis loop, see Figure 4a.

Upon further reduction of the modulation frequency, the peak current increases due to the longer time available for the capacitor to charge during the rising edge of the light intensity. However, during the falling edge of the light-intensity, the current drops below the level observed at 1 Hz for the same voltage. This behavior arises from the combined response of a large capacitor and an inductor in the circuit, the capacitor current during the capacitor charging slightly increases the peak current, but still the charge on the capacitor plates is not enough to turn the hysteresis into capacitive type. At the falling edge, the stored charge in the capacitor partially opposes the total current and thus current falls below than that observed at 1 Hz modulation frequency.

The presence of this large capacitor is even more evident when the modulation frequency is changed to 125 mHz. For this case the memristor current shows a transition from inductive hysteresis to capacitive hysteresis as shown in Figure S4. The mixed inductive- capacitive nature of our memristors at low scan rate, results in the complex evaluation of current voltage curves as a function of scanning speed.

In conclusion, by integrating a silicon solar cell with a volatile perovskite memristor, we have experimentally demonstrated and explained the complex dynamics of a coupled optoelectronic system. This synergetic coupling removes the traditional boundaries between power supply and computational unit, this work establishes a scalable foundation for autonomous, self-powered AI platforms. Such a system is uniquely positioned to perform light-driven neuromorphic computation, enabling edge-computing devices that can perceive and process information using only ambient or directed optical energy.

## Experimental Section/Methods

### Materials for Halide Perovskite Memristor

Methylammonium bromide (MABr, > 99.99%, Greatcellsolar), lead bromide (PbBr_2_, > 98.0%, TCI), N, N-dimethylformamide (DMF, anhydrous ≥ 99.8%, Avantorsciences), dimethyl sulfoxide (DMSO, ≥ 99.7%, Sigma-Aldrich), poly­(3,4-ethylenedioxythiophene)-poly­(styrenesulfonate) (PEDOT:PSS, Heraeus CleviosTM P VP AI 4083), and toluene (≥99.5%, Sigma-Aldrich) were used without further purification.

### Fabrication Methods for Halide Perovskite Memristor

A 1.4 M perovskite precursor solution was prepared by dissolving MABr and PbBr_2_ in a mixed solvent of DMF and DMSO (v/v %, 4:1). The MAPbBr_3_ solution was stirred thoroughly using a magnetic stirrer to ensure complete dissolution. Fluorine-doped tin oxide (FTO) substrates (2.5 cm^2^) were partially etched using zinc powder and a diluted hydrochloric acid (HCl) solution. The etched substrates were mechanically brushed to remove residuals and particles. They were cleaned via ultrasonication in deionized water (DIW) mixed with detergent, acetone, and isopropanol (IPA) sequentially for 15 min each, which is further dried with an air gun. The cleaned substrates were treated with ultraviolet ozone (UVO) for 30 min to improve surface wettability. PEDOT:PSS solution was filtered through a 0.45 μm PTFE-H syringe filter and spin-coated onto the UVO-treated substrates at 3000 rpm for 30 s, followed by annealing at 150 °C for 10 min on a hot plate. After cooling to room temperature, the filtered perovskite precursor solution through a 0.22 μm PTFE-H syringe filter was deposited via a two-step spin-coating process at 1000 rpm for 10 s and 4000 rpm for 30 s onto the PEDOT:PSS-coated FTO substrates. During the second step, 200 μL of toluene was dropped onto the spinning substrates as an antisolvent 10 s after the start of the step. The resulting films were then annealed at 100 °C for 30 min. Finally, an 85 nm-thick Au top electrode was deposited using a thermal evaporator to complete the full memristor structure.

### Characterization for a Coupled Halide Perovskite Memristor-Solar Cell System

Electrical characteristics were measured using an Autolab potentiostat (PGSTAT204). Light intensity of the LED source (AUT.LED.LDC530.S) was controlled by the LED driver (LD80294) connected with the FRA32 M module for the measurement of (I-ϕ) and (V-ϕ) curves.

## Supplementary Material



## Data Availability

The data presented here can be accessed at 10.5281/zenodo.18619370 (Zenodo) under the license CC BY 4.0 (Creative Commons Attribution 4.0 International).

## Notes

Illustration of solar cell and memristor coupling, theoretical model explaining memristor solar cell coupling, frequency dependent inductive and capacitive response of the memristor, endurance test of the complete device. (PDF)
