# Supplementary material for: Dynamical Symbiosis of Solar Cell and Memristor
Source: ACS Energy Lett. 2026 May 8;11(6):4512–7. doi: 10.1021/acsenergylett.6c00713 (PMC13270637; doi:10.1021/acsenergylett.6c00713)
Supplement: Supplementary file 1 [file nz6c00713_si_001.pdf]

Dynamical symbiosis of solar cell and memristor

Jitendra Kumar,[1,] So-Yeon Kim,[1] Gonzalo Rivera-Sierra,[1] Juan Bisquert[1,]**

[1]Instituto de Tecnología Química (ITQ), Consejo Superior de Investigaciones Científicas-Universitat Politècnica de València (CSIC-UPV), Valencia, Spain.

*Corresponding authors E-mail: jkumar2@itq.upv.es, jbisquer@itq.upv.es

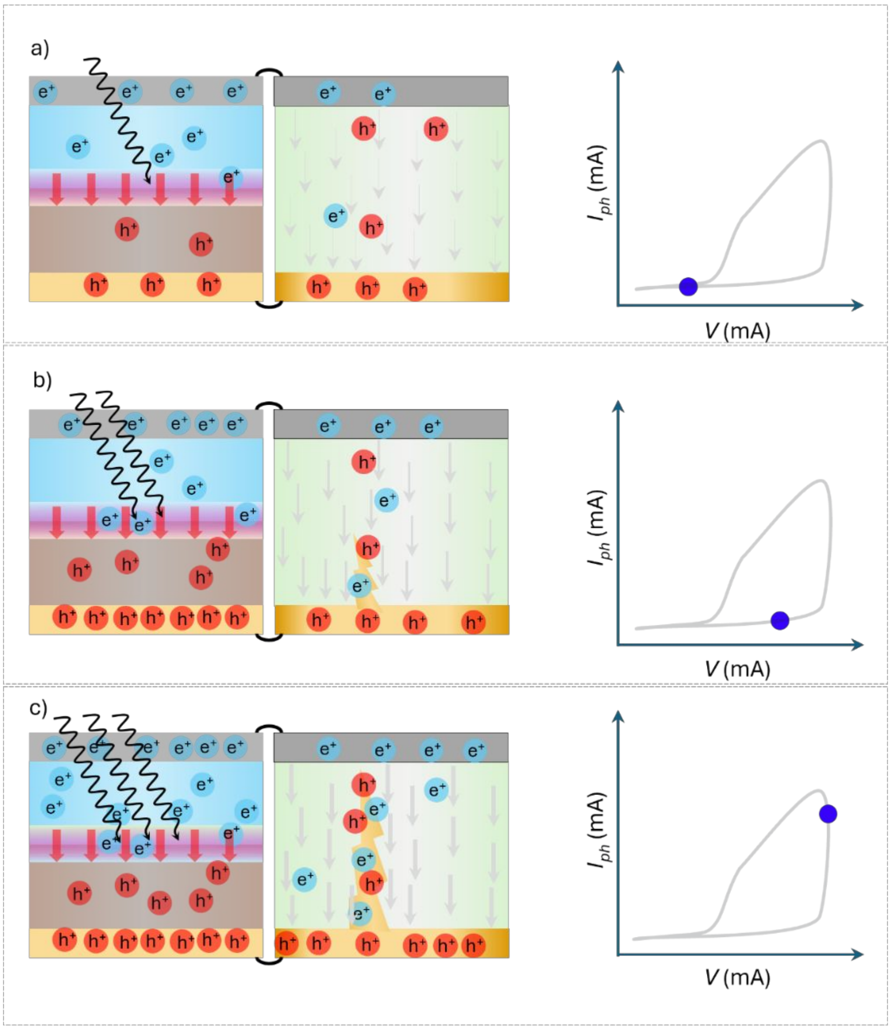

Figure S1. Schematic illustration of the different physical processes involved in resistive switching in coupled solar cell and memristor system.

Figure S1 illustrates the pathway from optical input to memristive state change in coupled memristor- solar cell system. Incident light on solar cell is absorbed and results in the generation of free electrons and holes. These free electrons and holes are separated by the electric field in the depletion region and collected on the respective electrode. This separation of electron and holes generate potential difference and is observed across the memristor. As the photovoltage increases with increasing light intensity it triggers the resistive switching of the memristor.

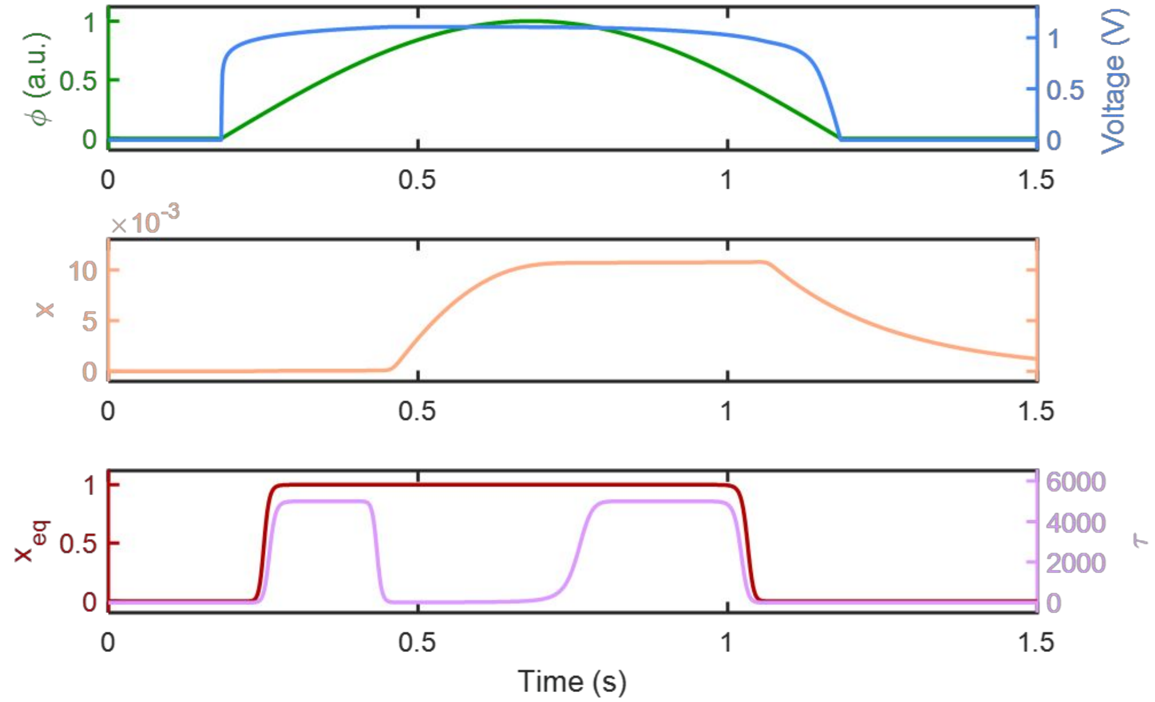

Figure S2. Evolution of memristor voltage, memristors' internal state variable, equilibrium state function and relaxation time constant as a function of light intensity.

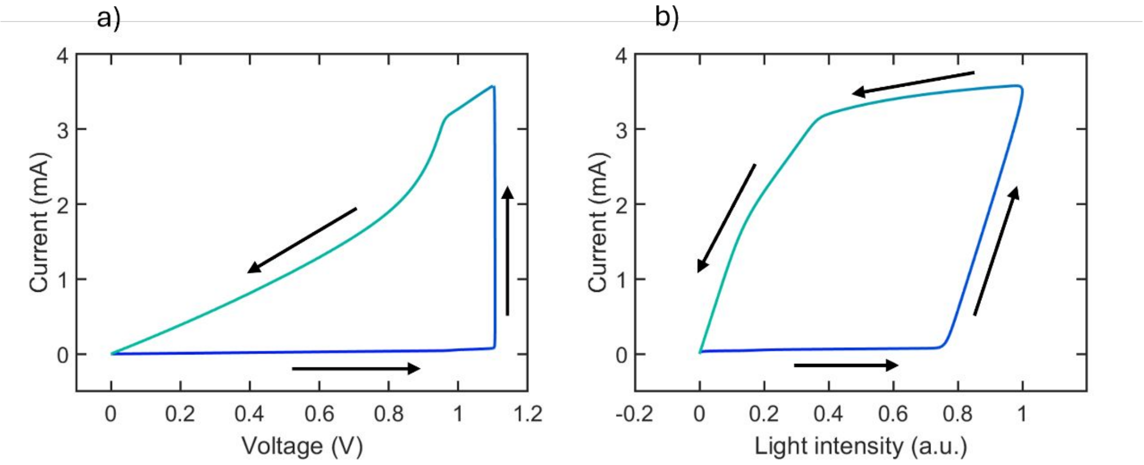

Figure S3. a) Current vs voltage and current vs light intensity characteristic of the coupled memristor solar cell.

Figures S2 and S3 show the simulated response of the coupled solar cell–memristor system within the CALM framework as described below. The model explains the observed volatile inductive hysteresis as the dynamic chase of the internal state variable toward its voltage-dependent steady-state value, with a relaxation time constant that is also voltage dependent.

Conductance-activated quasilinear memristor (CALM) model

The dynamics of the coupled system are described by a set of two first-order ordinary differential equations governing the cell voltage (V) and the internal memristive state variable (x).

Current voltage characteristics

The total current balance of the system, assuming a parallel capacitive component C_p, is given by:

$$C_p \frac{dV}{dt} = I_{solar}(V,\phi) - I_{mem}(V,x) \quad \ldots 1$$

where the solar cell current I_{solar} follows the ideal diode model under illumination

$$I_{solar} = I_{ph,0} \cdot \phi(t) - I_0 \cdot (\exp(aV) - 1) \quad \ldots 2$$

Here, $I_{ph,0}$ is short circuit current of the solar cell under the illumination intensity ϕ^*, $\phi(t)$ represents the time-dependent light intensity, and $a = q/(\eta_f k_B T)$ is the inverse thermal voltage scaled by the ideality factor η_f. The memristor current I_{mem} is defined by

$$I_{mem} = [g_L + (g_H - g_L)x] \quad \ldots 3$$

State variable dynamics

The evolution of the memristive state x is modeled using a voltage-dependent equilibrium state $x_{eq}(V)$ and voltage-dependent relaxation time constant $\tau(V)$, the differential equation for x is as follows[1]

$$\frac{dx}{dt} = \frac{x_{eq}(V) - x}{\tau(V)} \quad \ldots 4$$

The equilibrium state is a sigmoidal activation function

$$x_{eq} = \left[1 + \exp\left(-\frac{(V - V_x)}{V_m}\right)\right]^{-1} \quad \ldots 5$$

The effective time constant $\tau(V)$ is determined by the competition between SET and RESET processes, modeled as:

$$\tau(V) = \tau_n(V) - \tau_p(V) \quad \ldots 6$$

where $\tau_p(V)$ and $\tau_n(V)$ are given as follows:

$$\tau_p(V) = \frac{t_{max} - t_{min,p}}{1+\exp\left(-\left(\frac{V-V_{on}}{V_p}\right)\right)} \qquad \ldots 7$$

$$\tau_n(V) = \frac{t_{max} + t_{min,n} \cdot \exp\left(-\left(\frac{V-V_{off}}{V_n}\right)\right)}{1+\exp\left(-\left(\frac{V-V_{off}}{V_n}\right)\right)} \qquad \ldots 8$$

List of model parameters

η_f	3.19
a	12.07
I_0	1.61 × 10^{-8} A
$I_{ph,0}$	13.38 × 10^{-3} A
C_p	1 × 10^{-19} F
g_L	4.5 × 10^{-5} S
g_H	0.3 S
V_x	1 V
V_m	5 mV
V_n	5 mV
V_p	1 mV
V_{on}	1.1 V
V_{off}	1.01 V
t_{max}	5 × 10^3 s
$t_{min,n}$	0.2 s
$t_{min,p}$	1 ms

At low frequency the coupled system shows the presence of both inductive and capacitive dynamics. As shown in Figure S4 when the light modulation frequency is reduced from 500 mHz to 125 mHz the current shows a transition from inductive loop ($V < 1.05$ V) to capacitive loop ($V > 1.05$ V).

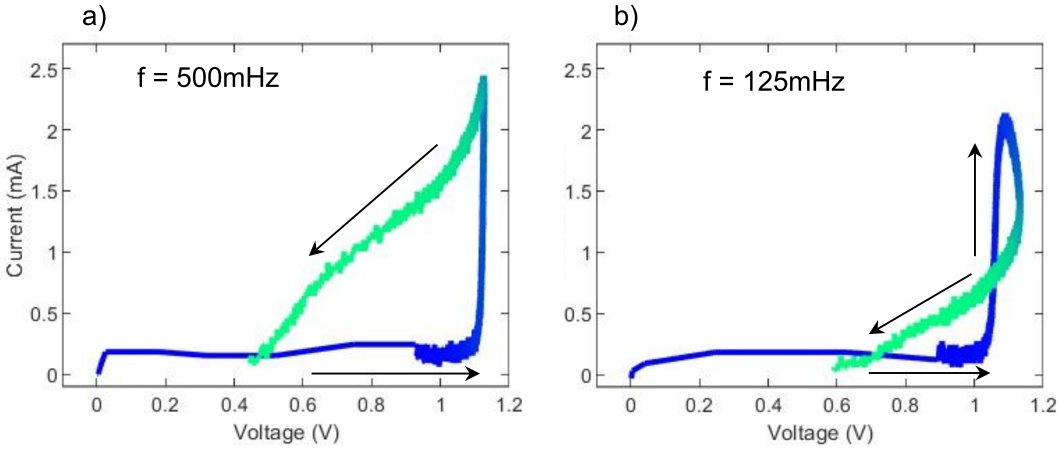

Figure S4. IV curve of coupled memristor-solar cell system. a) at 500 mHz modulation frequency of the light pulse b) at 125 mHz modulation frequency of the light pulse.

Device endurance and retention time

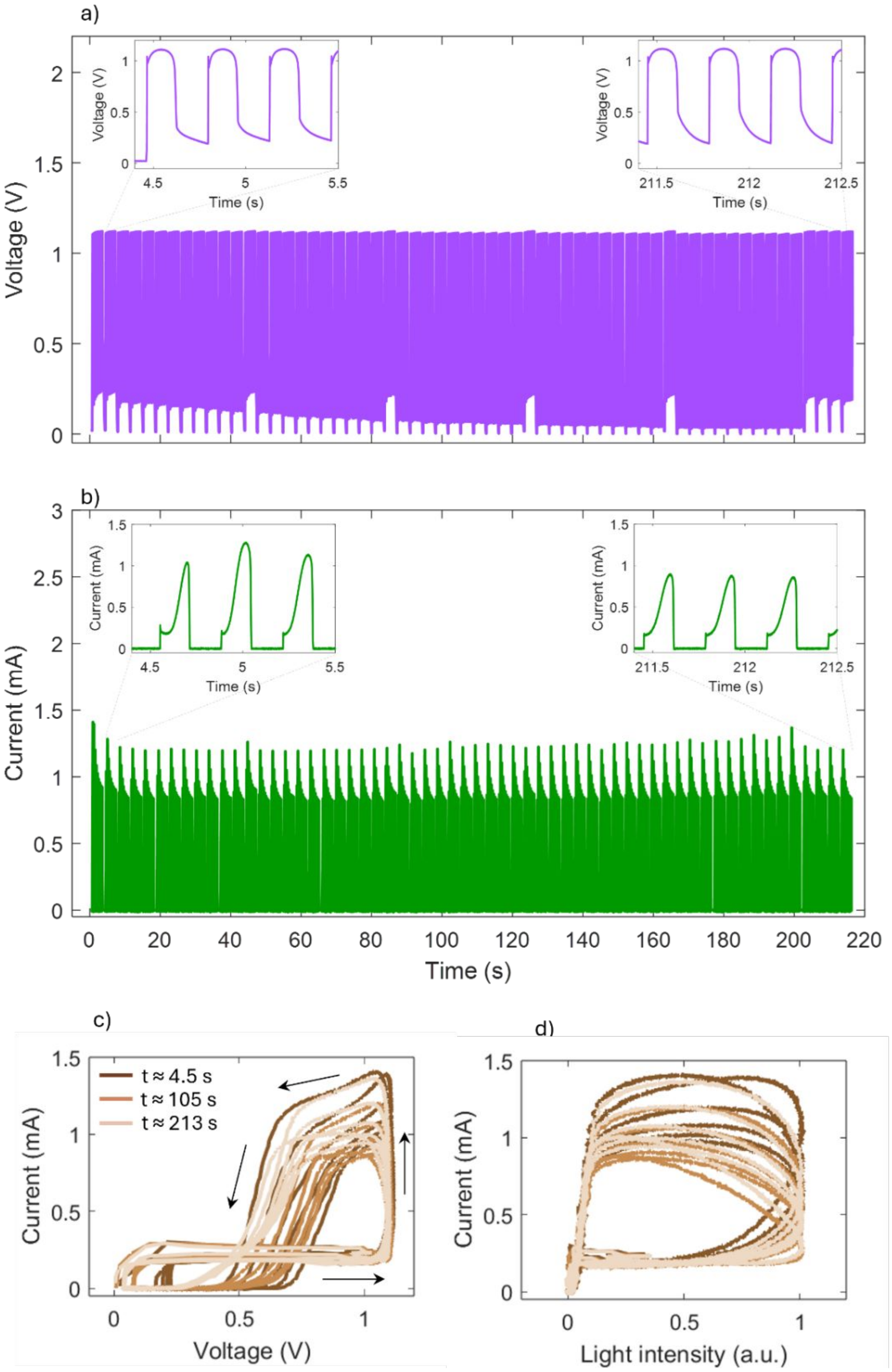

Figure S5. Device endurance test under the repeated applied pulses of light.

The endurance of the coupled system was evaluated by applying a total of 1080 light pulses. Optical stimulation was generated by modulating the light intensity as a sinusoidal waveform with a frequency of 3 Hz. As shown in Figure S5, the device exhibits no operational failure after 1080 cycles. Figure S5a presents the voltage response recorded during the first 540 light pulses, while Figure S5b shows the corresponding current response measured during the same endurance test. The IV curve in Figure S5c is derived from the current and voltage data shown in panels b and a, respectively, by correlating these 2 datasets by matching the corresponding light intensity. Since, this plots is reconstructed from two separate measurement traces and aligned through the applied light intensity; therefore, it serves as representative correlation rather than directly measured simultaneous IV. Figure 5d shows the light intensity vs current as measured in panel b.

Reference(s)

(1) Bou, A.; Gonzales, C.; Boix, P. P.; Vaynzof, Y.; Guerrero, A.; Bisquert, J. Kinetics of Volatile and Nonvolatile Halide Perovskite Devices: The Conductance-Activated Quasi-Linear Memristor (CALM) Model. *J. Phys. Chem. Lett.* **2025**, *16* (1), 69–76. https://doi.org/10.1021/acs.jpclett.4c03132.
